# A phospho-proteomic study of cetuximab resistance in *KRAS/NRAS/BRAF*^*V600*^ wild-type colorectal cancer

**DOI:** 10.1007/s13402-021-00628-7

**Published:** 2021-08-30

**Authors:** Alexandros Georgiou, Adam Stewart, Georgios Vlachogiannis, Lisa Pickard, Nicola Valeri, David Cunningham, Steven R. Whittaker, Udai Banerji

**Affiliations:** 1 Division of Cancer Therapeutics, The Institute of Cancer Research, Sycamore House, Downs Road, London, SM2 5PT UK; 2 Division of Molecular Pathology, The Institute of Cancer Research, Sycamore House, Downs Road, London, SM2 5PT UK; 3 Division of Clinical Studies, The Institute of Cancer Research, Sycamore House, Downs Road, London, SM2 5PT UK; 4 Department of Medicine, The Royal Marsden NHS Foundation Trust, Sycamore House, Downs Road, London, SM2 5PT UK

**Keywords:** Colorectal cancer, Cetuximab, Proteomics, Phospho-proteomics, Signalling adaptations, Resistance mechanisms

## Abstract

**Purpose:**

We hypothesised that plasticity in signal transduction may be a mechanism of drug resistance and tested this hypothesis in the setting of cetuximab resistance in patients with *KRAS/NRAS/BRAF*^*V600*^ wild-type colorectal cancer (CRC).

**Methods:**

A multiplex antibody-based platform was used to study simultaneous changes in signal transduction of 55 phospho-proteins in 12 *KRAS/NRAS/BRAF*^*V600*^ wild-type CRC cell lines (6 cetuximab sensitive versus 6 cetuximab resistant) following 1 and 4 h in vitro cetuximab exposure. We validated our results in CRC patient samples (n = 4) using ex vivo exposure to cetuximab in *KRAS/NRAS/BRAF*^*V600*^ cells that were immunomagnetically separated from the serous effusions of patients with known cetuximab resistance.

**Results:**

Differences in levels of phospho-proteins in cetuximab sensitive and resistant cell lines included reductions in phospho-RPS6 and phospho-PRAS40 in cetuximab sensitive, but not cetuximab resistant cell lines at 1 and 4 h, respectively. In addition, phospho-AKT levels were found to be elevated in 3/4 patient samples following ex vivo incubation with cetuximab for 1 h. We further explored these findings by studying the effects of combinations of cetuximab and two PI3K pathway inhibitors in 3 cetuximab resistant cell lines. The addition of PI3K pathway inhibitors to cetuximab led to a significantly higher reduction in colony formation capacity compared to cetuximab alone.

**Conclusion:**

Our findings suggest activation of the PI3K pathway as a mechanism of cetuximab resistance in *KRAS/NRAS/BRAF*^*V600*^ wild-type CRC.

**Supplementary Information:**

The online version contains supplementary material available at 10.1007/s13402-021-00628-7.

## Introduction

Cetuximab is a monoclonal antibody that targets the epidermal growth factor receptor (EGFR). As a single agent and in combination with cytotoxic agents, cetuximab has been shown to improve overall survival, when compared to placebo or standard of care, in patients with metastatic *KRAS* and *NRAS* exon 2–4 wild-type (WT) colorectal cancer (CRC) [1–3]. However, even in patients with *KRAS* WT tumours, with resistance to standard of care chemotherapy, the clinical efficacy of cetuximab is modest, with a monotherapy radiological response rate of 20% [1]. Evolutionary changes leading to the selection of the “fittest” *RAS* mutant resistant clones and other genetic aberrations such as *PIK3CA, BRAF* and extracellular *EGFR* domain mutations, *HER2* amplifications and *ALK/ROS1/NTRKs/RET* fusions have also been suggested as mechanisms of anti-EGFR antibody resistance [4–7]. In addition to genetic aberrations, compensatory signalling crosstalk (for example between EGFR and MET receptor or EGFR with other ErbB family receptors) have been suggested to contribute to the development of cetuximab resistance [6, 8].

Phospho-proteomic changes following exposure to targeted therapies can be regarded as a quantifiable surrogate of adaptive signalling. To date, no studies have been reported on the profiling of phospho-proteomic changes upon exposure to cetuximab in *KRAS/NRAS/BRAF*^*v600*^ WT CRC cells. We hypothesised that studying re-wiring of signal transduction in response to cetuximab may provide novel insights into mechanisms of drug resistance in the setting of *RAS*/*BRAF*^*V600*^ WT CRC. To test this hypothesis, we utilised 12 *KRAS/NRAS/BRAF*^*V600*^ WT cell lines, representing both cetuximab sensitive and resistant cells, and patient-derived samples that were isolated from serous effusions of patients with *KRAS/NRAS/BRAF*^*V600*^ WT CRC, who were resistant to cetuximab. We next used a multiplex antibody-based proteomic platform to quantify simultaneous changes in 55 phospho-proteins, following 1 and 4 h of cetuximab exposure. Our phospho-proteomic panel included 26 receptor and non-receptor tyrosine kinases as well as phospho-proteins that function within pathways that are known to be key in CRC, including the MAPK, PI3K, JAK-STAT and Wnt/β-Catenin pathways. All findings of interest were validated and further investigated.

## Methods

### Cell lines, tissue culture and anti-cancer agents

The SNUC1, CACO2 and LIM1215 cell lines were purchased from the American Tissue Culture Collection (ATCC) and the C10, C70 and HCA24 cell lines from Public Health England (PHE). The NCIH508, HCA46, SW48 and COLO320 cell lines were kindly donated by other laboratories within the Institute of Cancer Research (ICR) London. OXCO2 and DiFi were kindly donated by Professor Alberto Bardelli’s team, Candiolo Cancer Institute, Turin, Italy. The known mutations in the cell line panel are described in Supplementary Table 1. Cetuximab was kindly provided by Merck Serono Ltd., UK, an affiliate of Merck KGaA, Darmstadt, Germany. GDC0941 and AZD2014 were purchased from Selleck Chemicals.

### Isolation of cancer cells from serous effusions of patients with ***KRAS/NRAS/BRAF***^***V600***^ WT CRC

Up to 1000 ml ascites or pleural fluid was collected from patients at the time of therapeutic drainage after which cancer cells were immunomagnetically separated on the same day using previously published methods [9, 10].

### Quantification of phosphoproteins

#### Luminex 200 magnetic bead suspension array

The material and methods used have been described previously [9]. All phospho-proteins that were quantified are listed in Supplementary Fig. 1. In brief, cells were equally seeded in 25 cm^2^ flasks and cultured to 70% confluency. Next, the cells were exposed to cetuximab or left untreated for 1 and 4 h. To closely model the clinical setting, the concentration of cetuximab used (306 µg/ml) was equal to the peak serum concentration (C_max_) that cetuximab has been reported to reach in patients’ plasma in early phase clinical trials [11]. Since cetuximab protein binding in plasma is negligible, the C_max_ concentration in cell culture was not modified [12]. After 1 or 4 h exposure, cells were lysed in 43–040 (MerckMillipore) supplemented with protease (I3786, Sigma Aldrich, UK) and phosphatase (4,906,845,001, Sigma Aldrich) inhibitors. Ten μg protein was loaded in each well of a 96-well plate and processed as per manufacturers' protocol. The quality control measures for our antibody-based multiplex platform have been previously described [9].

**Fig. 1:**
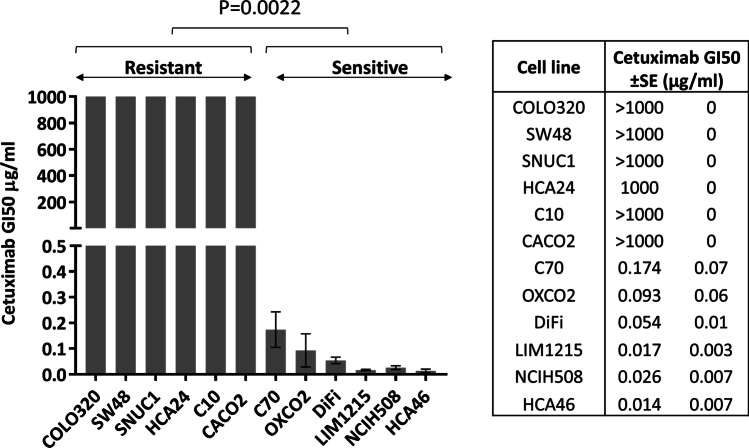
*KRAS/NRAS/BRAF*^*V600*^ WT CRC cell line sensitivity to cetuximab. Mean GI_50_ values are shown ± standard error (n = 3); data are representative of 3 independent experiments. Six cell lines (COLO320, SW48, SNUC1, HCA24, C10, CACO2) were classified as cetuximab resistant as a GI_50_ was not reached at the highest cetuximab concentration used (1000 µg/ml). Six cell lines (C70, OXCO2, DiFi, LIM1215, NCIH508, HCA46) were classified as sensitive with a mean GI_50_ 0.063 (± 0.025) µg/ml. The difference between cetuximab sensitive and resistant cell lines was statistically significant (Mann Whitney test *p* = 0.0022)

#### Quantification of proteins using Western blot analysis

Western blot analysis was carried out using standard techniques. The concentrations of the antibodies used are documented in Supplementary Table 2.

### Cell proliferation assays

#### Sulforhodamine B (SRB) and CellTiter-Blue (CTB) assays

Cells were seeded in 96-well plates for 24 h before being treated with serial dilutions of the desired inhibitor, or vehicle control. The range of cetuximab concentrations used for the cell lines was 0.008 to 1000 µg/ml. After 72 h, cells were fixed using 10% trichloracetic acid, washed with double distilled water, air-dried and stained using 0.4% SRB in 1% acetic acid for 20 min. SRB was solubilized using 10 mM Tris–HCl and the absorbance was read at 570 nm wavelength using a Titertek Multiscan MCC/340 MKII plate reader. For non-adherent cell lines (SNUC1) a CellTiter-Blue assay was used. For this, cells were seeded and treated in same way as per SRB assay. After 72 h CellTiter-Blue reagent was added at a 1:10 dilution and incubated for 4 h. Next, fluorescence was quantified using an EnVison plate reader. GI_50_ values were calculated using GraphPad prism 8.0.

#### Clonogenic assay

Cells were seeded into 12-well plates (2.5 × 10^3^ to 7.5 × 10^3^ /well) and 24 h later inhibitors were added to the cells and incubated for 14 days. A concentration of GDC0941 and AZD2014 of 100 nM was chosen based on preliminary experiments that confirmed downstream signalling inhibition at the chosen dose (e.g. pPRAS40 and pS6, see Supplementary Fig. 2). Next, cells were fixed with 4% formaldehyde/PBS for 20 min and stained with 0.5% crystal violet in 70% ethanol. Finally, plates were imaged using a GelCount (Oxford Optronix) system.

**Fig. 2:**
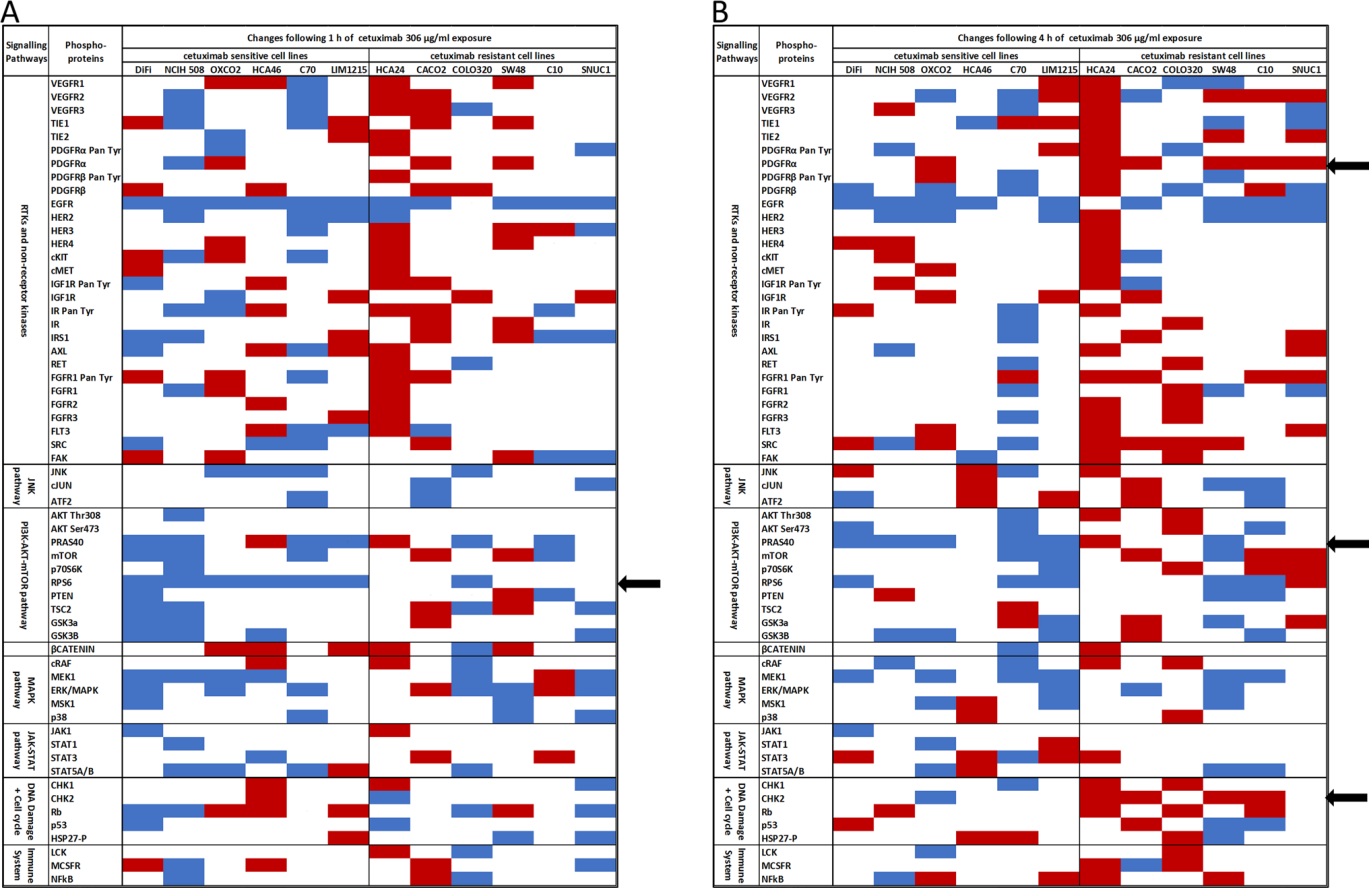
Changes in phospho-protein levels in cetuximab sensitive and resistant *KRAS/NRAS/BRAF*^*V600*^ WT CRC cell lines**.** Phosphoprotein changes following 1 (**a**) and 4 (**b**) h cetuximab exposure. In red phospho-proteins whose expression is increased, defined as having a cetuximab treated value that was at least 2 S.D. above the mean of three untreated controls. In blue, phospho-proteins whose expression is decreased, defined as a cetuximab treated value of at least 2 S.D. below the mean of controls. In white, phospho-proteins that were unchanged compared to controls. The black arrows indicate the phospho-protein changes that are statistically significant after Benjamini–Hochberg (BH) correction (*p* < 0.05). Phosphorylated RPS6 expression was statistically more likely to be downregulated in cetuximab sensitive cell lines following 1 h cetuximab exposure. At 4 h exposure phospho-PRAS40 was more likely to be decreased in sensitive cell lines, whereas pPDGFRα and pCHK2 were more likely to be increased in cetuximab resistant cell lines

### Statistical tests and interpretation of multiplex phospho-proteomics data

For each of the cell lines used for both the 1 and 4 h experiments, three control samples and one cetuximab sample were used. All phospho-proteomic data were normalised to GAPDH. The mean and standard deviation (SD) of the median fluorescent intensity (MFI) were calculated for the three controls. For each cell line, when the cetuximab treated value was > 2 SDs above the mean of the controls it was classified as ‘increased’ and when it was < 2 SDs below the mean of the controls it was classified as ‘decreased’. If the cetuximab treated value was within 2 SDs of the mean of the controls it was considered unchanged. We chose to use this method for our cell line screens, as we did not validate the linearity of the absolute changes in phospho-proteins in our assays.

For the ex vivo patient sample experiments, due to limited cell numbers, both 1 and 4 h experiments were not possible. Therefore, we were only able to carry out 1 h exposure experiments. The ex vivo experiments were otherwise conducted in the same way as the cell line experiments. For each patient sample we included 3 untreated controls and one drug exposed sample. Then the ratios of the cetuximab treated to untreated controls were calculated using the following equation: ratio of change = MFI of cetuximab treated sample / mean MFI of three controls. A phospho-protein with a ratio > 1 meant that there was an increase in phospho-protein expression compared to the controls, and the reverse was true if the ratio was < 1.

Differences in GI_50_ between sensitive and resistant cell lines were analysed using a Mann Whitney test (GraphPad Prism 8.0). Statistical differences in phospho-proteomic changes following exposure to cetuximab between cetuximab sensitive and resistant cell lines were studied using Binary Logistic regression corrected for False Discovery Rate (FDR) via the Benjamini–Hochberg (BH) procedure. Differences between readouts of clonogenic assays of cells treated with cetuximab, GDC0941, AZD2014 or the combination were analysed using *t* tests (GraphPad Prism 8.0).

## Results and discussion

Six cell lines (COLO320, SW48, SNUC1, HCA24, C10 and CACO2) were classified to be cetuximab resistant, as a GI_50_ was not reached at any concentration, with the highest cetuximab concentration used being 1000 µg/ml. Another six cell lines (C70, OXCO2, DiFi, NCIH508, LIM1215 and HCA46) were classified as cetuximab sensitive; mean (± S.E) cetuximab GI_50_ 0.063 (± 0.025) µg/ml. The cetuximab GI_50_ difference between sensitive and resistant groups was significant (*p* = 0.0022, Fig. 1).

A number of phospho-proteins was found to be differentially regulated in the cetuximab sensitive versus resistant cell lines following 1 and 4 h exposure to 306 µg/ml cetuximab, a concentration that was clinically achievable in C_max_ concentration trials [11]. Binary logistic regression and adjustments for multiple testing were applied to test for changes that were statistically significant (Fig. 2). A decrease in phospho-RPS6 levels was statistically more likely to occur in cetuximab sensitive cell lines following 1 h of cetuximab exposure (*p* = 0.04). After a 4 h exposure another closely related PI3K pathway phospho-protein, phospho-PRAS40, was more likely to be decreased in cetuximab sensitive compared to resistant cell lines (*p* = 0.04). Conversely, phospho-PDGFRa and phospho-CHK2 were found more likely to be increased in cetuximab resistant versus sensitive cell lines (*p* = 0.04 and *p* = 0.03, respectively). Interestingly, phospho-EGFR levels were reduced in both cetuximab sensitive and resistant cell lines at a comparable level, which suggests that cetuximab continued to inhibit its intended target and that differences in sensitivity were possibly related to downstream signalling.

**Fig. 3:**
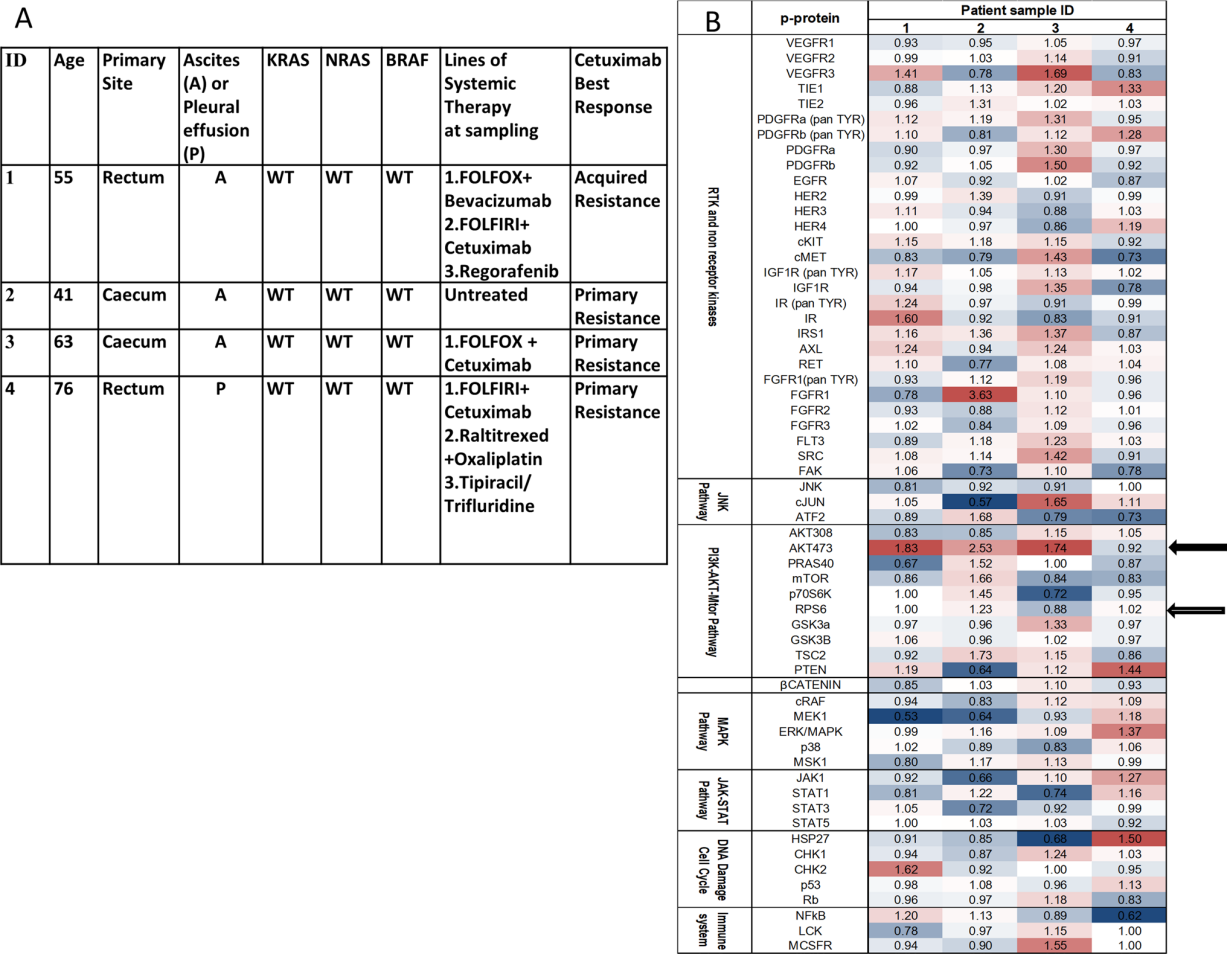
Clinical features and phospho-proteomic changes in cancer cells isolated from ascites/pleural effusions. **a**: Clinicopathological characteristics of patients at the time of sample collection, including the lines of systemic therapy received. Mutation data are based on standard diagnostics carried out on primary tumour samples. **b**: Cells were immunomagnetically isolated from patients’ serous effusions and were treated ex vivo with 306 µg/ml cetuximab or left untreated (n = 4) for 1 h. Mean ratios of change (cetuximab treated to untreated controls) for all phospho-proteins quantified in 4 *KRAS/NRAS/BRAF*^*V600*^ wild-type CRC patient samples are shown. A ratio > 1 (in red) suggests increase in phospho-protein expression in the cetuximab treated cells compared to the mean of controls and a ratio < 1 (blue) the reverse. A lack of reduction in phospho-RPS6 in 3 of 4 patient samples following exposure to cetuximab was observed (lower arrow). In addition, an increase in phospho-AKT Ser473 levels was observed in 3 of 4 cell patient samples (upper arrow) following exposure to cetuximab, suggesting continued activation of the PI3K pathway

In 4 patient-derived samples, cells were immunomagnetically isolated from serous effusions. The clinical history of all 4 patients was suggestive of resistance to cetuximab (Fig. 3a). Changes in all phospho-proteins following cetuximab exposure are presented as ratios of changes compared to the untreated controls (Fig. 3b). The 1 h patient sample and cetuximab resistant cell line results were largely concordant. For example, a lack of reduction in phospho-RPS6 in 3 of 4 patient samples following exposure to cetuximab was seen in both resistant cell lines and patient samples. Interestingly, an increase in phospho-AKT^Ser473^ levels was seen in 3 out of 4 patient samples following exposure to cetuximab, suggesting continued activation of the PI3K pathway.

**Fig. 4:**
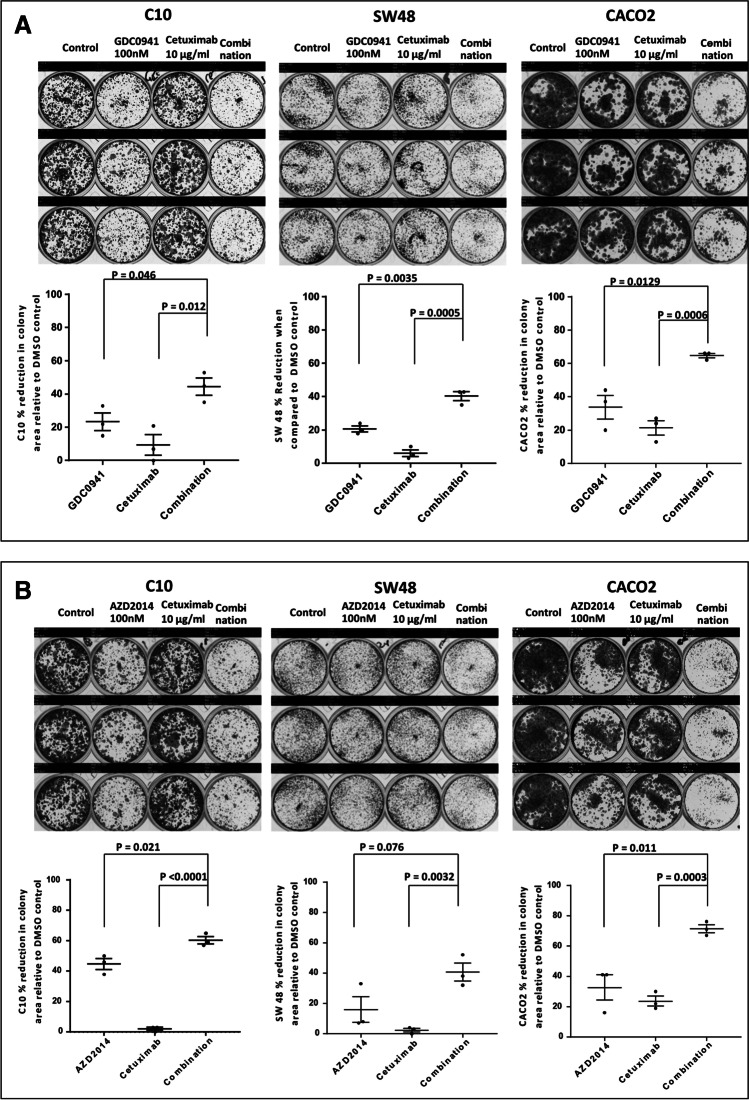
Growth inhibition caused by combination of cetuximab and PI3K pathway inhibitors. Two-week clonogenic assays in 3 cetuximab resistant cell lines, C10, SW48, CACO2. All cells were treated with 10 µg/ml cetuximab. **a:** Combination with 100 nM GDC-0941; **b:** Combination with 100 nM AZD2014. Images are representative of 3 repeats. The mean (± S.E.) reduction of total area density in relation to the control is shown (n = 3) for each treatment condition, along with *t*-test analysis

In view of the findings in the cell lines which showed that phospho-RPS6 and phospho-PRAS40 levels were not reduced in response to cetuximab in the cetuximab resistant cell lines, when compared to the cetuximab sensitive cell lines, and the finding that phospho-AKT^Ser473^ was increased in 3 out of 4 CRC samples exposed to cetuximab ex vivo for 1 h, we decided to explore the effects of the addition of PI3K pathway inhibitors to cetuximab in cetuximab resistant cell lines. Three cetuximab resistant cell lines (C10, SW48 and CACO2) were exposed to 100 nM pictilisib (also known as GDC0941, a PI3K inhibitor) and vistusertib (also known as AZD2014, a m-TORC1/2 inhibitor). Inhibition of phospho-RPS6 and phospho-PRAS40 was confirmed at these concentrations. We also conducted single agent and combination therapies and subsequently applied Western blot analyses to SW48 and CACO2 cell lines (Supplementary Fig. 2). Downregulation of the PI3K pathway and induction of apoptosis in the combination experiments in excess of that seen with single agents was modest and was not consistent across all cell lines. We also investigated the effects of the combination of 10 µg/ml cetuximab and the two PI3K pathway inhibitors using 14 day clonogenic assays. We observed a statistically significant higher inhibition of colony forming capacity following the addition of PI3K pathway inhibitors to cetuximab, when compared to single agent exposure, in all three cetuximab resistant cell lines (Fig. 4).

The role of *KRAS/NRAS* mutations in cetuximab resistance is well-documented and they lead to continued signalling downstream of EGFR through the PI3K and MEK-ERK pathways [13, 14]. The response rates of cetuximab monotherapy in patients with chemotherapy resistant *KRAS* WT CRC is, however, only ~ 20% and therefore there remains an unmet need to improve their clinical outcome [1]. We aimed to investigate the role of signalling adaptations by determining sensitivity to cetuximab in *KRAS/NRAS/BRAF*^*V600*^ WT CRCs. This is the first report of an antibody-based phospho-proteomic screen of dynamic changes in signal transduction ex vivo in cancer cells derived from patients with cetuximab resistant tumours. However, we had access to only a limited number of samples. Indeed, current literature suggests that patients with *KRAS* mutant tumours are more likely to develop serous effusions, a phenomenon that we also observed before [15, 16]. This may have contributed to the relatively small number of samples of cells isolated from patients with *KRAS/NRAS/BRAF*^*V600*^ WT tumours that we were able to include.

Previous studies that investigated adaptations in signalling pathways as a mechanism of resistance to cetuximab suggested activation of receptor tyrosine kinases such as HER3 and non-receptor kinases such as SRC [17, 18]. While in our screen phospho-HER3 and SRC levels were increased in a number of cetuximab resistant cell lines following 1 h and 4 h of cetuximab exposure, respectively, the results were not statistically significant upon correction for multiple testing. This may at least in part have been due to differences between the cell lines and patient samples used. For example, in studies identifying SRC as a regulator of cetuximab sensitivity/resistance, *KRAS* mutant cell lines such as HCT116 and HT29 were used [18]. Instead in our data set, when corrected for multiple testing, only 4 changes were significant of which two (phospho-RPS6 and phospho-PRAS40) were related to the PI3K pathway. We speculate that in different cetuximab resistant cells, different receptor and non-receptor tyrosine kinases may be activated, with co-activation of several receptors being likely. This may subsequently result in the observed PI3K pathway upregulation. Indeed 3/4 of our samples isolated from CRC patients with established cetuximab resistance showed increases in phospho-AKT levels when exposed ex vivo to cetuximab. We subsequently investigated the effects of the combination of PI3K pathway inhibitors with cetuximab in three cetuximab resistant cell lines and observed additional growth inhibition when compared to single agent treatment activity.

Activation of the PI3K pathway in *KRAS* mutated CRC has been reported before [9, 19], but little is known about this in the *KRAS/NRAS/BRAF*^*V600*^ WT setting [20]. Factors that have been thought to be important in a *KRAS/BRAF*^*V600*^ WT setting include CCR7 expression [21] and mutations in genes regulating the PI3K pathway [22]. In contrast, another study has suggested that signalling through the PI3K pathway may confer a better response to anti-EGFR pathway treatment in this setting [23].

Other groups have previously suggested that PI3K pathway inhibitors such as PI3K, AKT and m-TOR inhibitors may be effective when combined with cetuximab, which is in agreement with our current data [24, 25]. Clinical trials have explored the combination of P13K pathway inhibitors (e.g. PX-886) with cetuximab in the *KRAS* WT setting without any improvement in clinical efficacy, but with increased toxicity in the combination over cetuximab alone [26]. On the other hand, a clinical trial that investigated addition of the m-TOR inhibitor everolimus to cetuximab and irinotecan in 43 patients suggested that the combination was clinically active and that it should be further developed [27]. Combinations of PI3K pathway inhibitors and cetuximab in the *KRAS/NRAS/BRAF*^*V600*^ WT setting should be explored in vivo along with biomarkers (e.g. PTEN loss) that may further enrich clinical benefit. These efforts may lead to combinations which are clinically actionable.

Our phospho-proteomic screen, though not extensive, did uncover actionable signalling nodes from multiple different signal transduction pathways such as receptor tyrosine kinases (RAS-RAF-MEK, PI3K-AKT-m-TOR), angiogenesis receptors (JAK-STAT, WNT) and DNA damage repair pathways (supplementary Fig. 1). It is of interest to note that upon correction for multiple testing it was mainly the PI3K pathway that showed differences in signalling between cetuximab sensitive and resistant lines at early time points. This offers insights into differences in dependencies on signal transduction pathways between cetuximab resistant and sensitive *KRAS/NRAS/ BRAF*^*V600*^ WT CRC.

We also report dynamic signalling patterns in freshly isolated cancer cells from serous effusions in patients with *KRAS/NRAS/BRAF*^*v600*^ WT CRC who were resistant to cetuximab therapy. Though the number of samples was small, it was encouraging to see concordance between activation of the PI3K pathway between the cell lines and patient samples in the assays studying early changes in dynamic signal transduction.

We acknowledge some limitations of our work. All cell lines were cultured to 70% confluency before 1 or 4 h exposure to control or cetuximab. This may have led to differences in the actual cell numbers seeded between cell lines at the time of cetuximab dosing. This was compensated by seeding equal amounts of protein in our phospho-proteomic experiments for all cell lines and patient samples used. Moreover, all phospho-proteomic results were adjusted for loading using GAPDH. In our 25 cell line 1 and 4 h cetuximab exposure experiments and in our 1 h ex vivo patient sample experiments, one cetuximab exposed and three untreated control samples were used. This was compensated by our strict definition of a ‘hit’ in our phospho-proteomic analysis. A significantly altered phospho-protein expression was defined as a treated value of 2 standard deviations above or below the mean of the three controls. Moreover, when comparing cetuximab sensitive and resistant cells, our statistical analysis for significance included adjustment for multiple testing. Lastly, the patient sample genomic data were derived from analysis of the primary tumours and this was not confirmed in the malignant cells isolated from the serous effusions. We acknowledge that tumour heterogeneity and effects of previous treatments may have affected these genomic aberrations. Nevertheless, there is evidence to suggest that there is high genomic concordance between matched primary and metastatic CRC samples [28].

In conclusion, our phospho-proteomic study of acute re-wiring of signal transduction, following exposure to clinically relevant concentrations of cetuximab, in a panel of *KRAS/NRAS/BRAF*^*V600*^ WT cell lines and ex vivo in samples derived from patients with clinical resistance to cetuximab, strongly suggests continued activation of the PI3K pathway as a mechanism of resistance to cetuximab. Combinations of PI3K pathway inhibitors and cetuximab in the *KRAS/NRAS/BRAF*^*V600*^ WT setting should be explored in vivo, which may lead to combinations that are clinically actionable.

## Supplementary Information

Below is the link to the electronic supplementary material.

Phospho-proteomic screen overview and list of phospho-proteins that were quantified simultaneously. Phosphorylated tyrosine kinases in which a phospho-site is not specified were quantified using a MiliporeMerck MILLIPLEX RTK phosphorylation kit that utilised a pan-tyrosine secondary antibody. Phosphorylated: FGFR1-3, IR, IGF1R, PGFRα and β were quantified using the MILLIPLEX RTK phosphoprotein kit and also single-plex kits that quantified the phospho-proteins specified (PPTX 148 kb)

Western Blotting for cetuximab and GDC0941 (A) and AZD2014 (B) combinations in cetuximab resistant cell lines. Cetuximab resistant cell lines: SW48 and CACO2 were treated with: DMSO control, AZD2014 or GDC0941 100 nM, cetuximab10 µg/ml or a combination of the two for 24 hr and 72 hr. Cell lysates were made and analysed by Western blotting for the indicated proteins. All antibodies were purchased from Cell Signalling, aside GAPDH that was purchased from Merck Millipore (see Supp Table 2) (TIF 1016 kb)

Supplementary file3 (DOCX 16 kb)

Supplementary file4 (DOCX 13 kb)

## Abbreviations

AKT: Serine-threonine protein kinase also called protein kinase B
ALK: Anaplastic lymphoma kinase
BRAF: V-raf murine sarcoma viral oncogene homolog B1
CCR7: C–C chemokine receptor type 7
CHK: Checkpoint kinase
Cmax: The peak serum concentration
EGFR: Epidermal frowth factor receptor
ErbB: Family of proteins-contains four receptor tyrosine kinases, structurally related to the epidermal growth factor receptor
GI50: Growth inhibitory 50%
HER2: Human epidermal growth factor receptor 2
HER3: Human epidermal growth factor receptor 3
ICR: Nstitute of Cancer Research London UK
JAK: Janus Kinase
KRAS: Kirsten rat sarcoma viral oncogene
MAPK: Mitogen-activated protein kinas
MEK: Mitogen-activated protein kinase kinase
MET: MET proto-oncogene, receptor tyrosine kinase
MFI: Median fluorescent intensity
mTOR: Mammalian target of rapamycin
NRAS: Neuroblastoma ras viral oncogene homologue
NTRK: Neurotrophic-tropomyosin receptor kinase
PDGFR: Platelet growth factor receptor
PI3K: Phosphatidylinositol 3-kinase
PIK3CA: Phosphatidylinositol 3-kinase catalytic alpha polypeptide
PRAS40: He proline-rich Akt substrate of 40 kDa
ROS: Proto-oncogene tyrosine-protein kinase ROS
SD: Standard deviation
SRB: Sulforhodamine B
SRC: Proto-oncogene tyrosine-protein kinase Src
STAT: Signal transducer and activator of transcription protein
WT: Wild-type
