# Supplementary figures and images for: A phospho-proteomic study of cetuximab resistance in KRAS/NRAS/BRAFV600 wild-type colorectal cancer
Source: Cell Oncol (Dordr). 2021 Aug 30;44(5):1197–206. doi: 10.1007/s13402-021-00628-7 (PMC8516765; doi:10.1007/s13402-021-00628-7)

## Slide 1
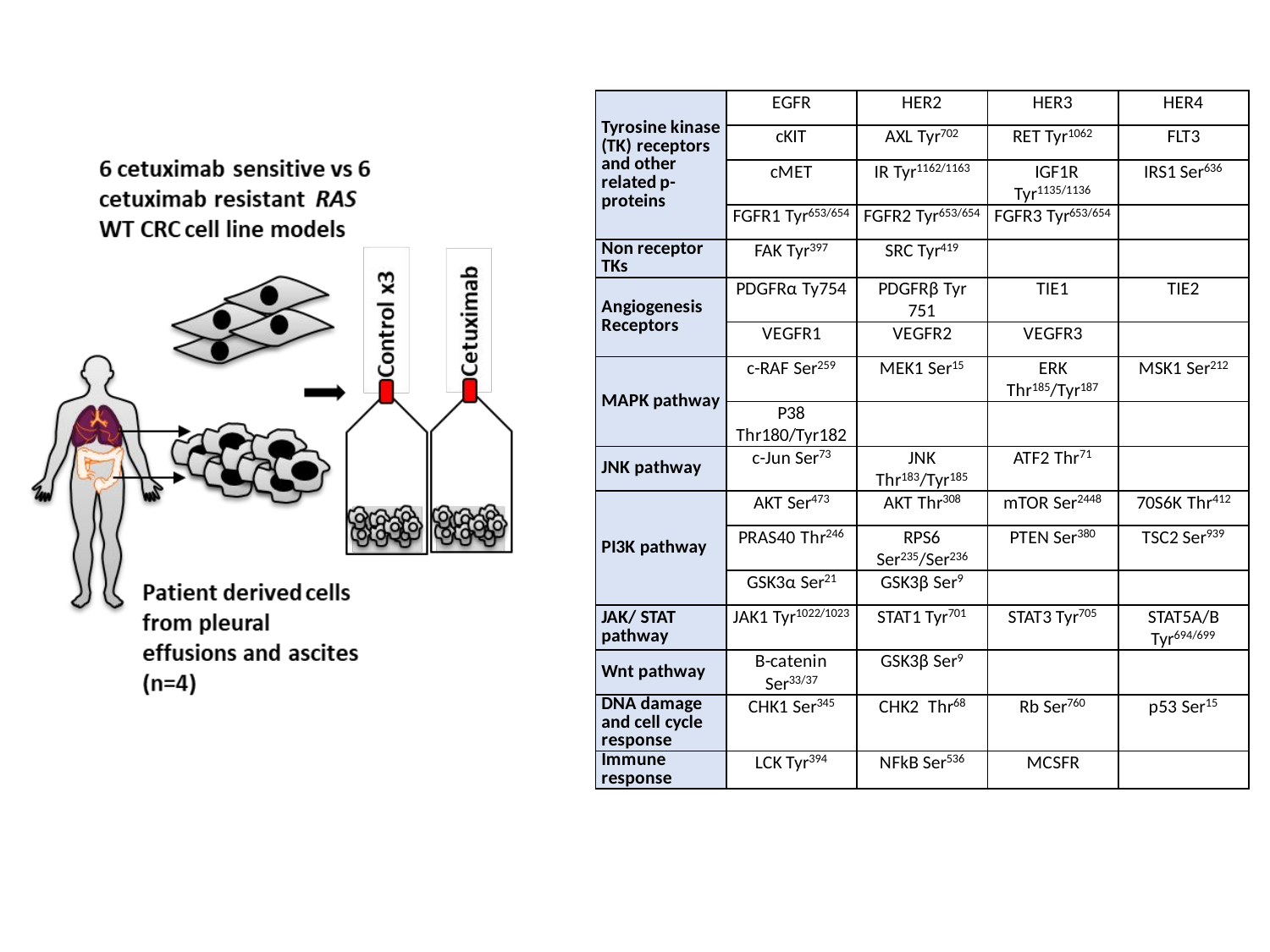

Supplement: Supplementary file 1 — Phospho-proteomic screen overview and list of phospho-proteins that were quantified simultaneously. Phosphorylated tyrosine kinases in which a phospho-site is not specified were quantified using a MiliporeMerck MILLIPLEX RTK phosphorylation kit that utilised a pan-tyrosine secondary antibody. Phosphorylated: FGFR1-3, IR, IGF1R, PGFRα and β were quantified using the MILLIPLEX RTK phosphoprotein kit and also single-plex kits that quantified the phospho-proteins specified (PPTX 148 kb) [file 13402_2021_628_MOESM1_ESM.pptx]

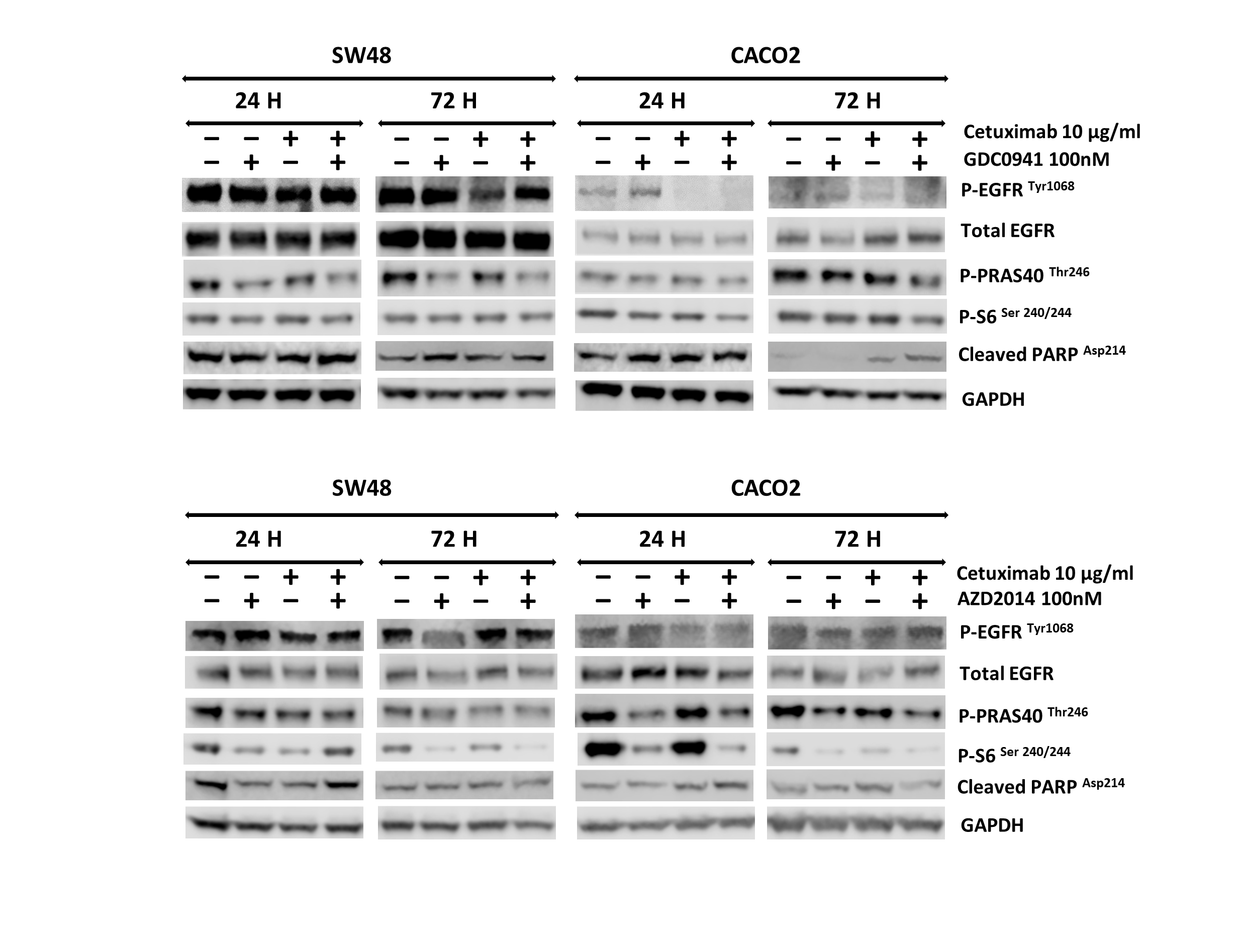

Supplement: Supplementary file 2 — Western Blotting for cetuximab and GDC0941 (A) and AZD2014 (B) combinations in cetuximab resistant cell lines. Cetuximab resistant cell lines: SW48 and CACO2 were treated with: DMSO control, AZD2014 or GDC0941 100 nM, cetuximab10 µg/ml or a combination of the two for 24 hr and 72 hr. Cell lysates were made and analysed by Western blotting for the indicated proteins. All antibodies were purchased from Cell Signalling, aside GAPDH that was purchased from Merck Millipore (see Supp Table 2) (TIF 1016 kb) [file 13402_2021_628_MOESM2_ESM.tif]
