# Supplementary material for: A phospho-proteomic study of cetuximab resistance in KRAS/NRAS/BRAFV600 wild-type colorectal cancer
Source: Cell Oncol (Dordr). 2021 Aug 30;44(5):1197–206. doi: 10.1007/s13402-021-00628-7 (PMC8516765; doi:10.1007/s13402-021-00628-7)
Supplement: Supplementary file 3 — Supplementary file3 (DOCX 16 kb) [file 13402_2021_628_MOESM3_ESM.docx]

# Supplementary Tables

| **Cell line** | **Source** | **Culture Media** | **EGFR** | **KRAS** | **NRAS** | **HRAS** | **BRAF** | **PIK3CA** | **APC** | **TP53** |
| --- | --- | --- | --- | --- | --- | --- | --- | --- | --- | --- |
| **CACO2** | ICR | MEM | WT | WT | WT | WT | WT | WT | Mut | Mut |
| **C10** | PHE | IMDM | X | WT | WT | WT | WT | WT | X | X |
| **C70** | PHE | IMDM | X | WT | WT | WT | WT | WT | X | X |
| **HCA24** | PHE | DMEM | X | WT | WT | WT | WT | WT | X | X |
| **COLO320** | ICR | RPMI1640 | WT | WT | WT | WT | WT | WT | Mut | Mut |
| **DiFi** | CCI | DMEM/F12 | WT | WT | WT | WT | WT | WT | Mut | Mut |
| **HCA46** | ICR | DMEM | X | WT | WT | WT | WT | Mut | X | X |
| **LIM1215** | ATCC | RPMI-supp | WT | WT | WT | WT | WT | WT | Mut | WT |
| **NCIH508** | ICR | RPMI 1640 | WT | WT | WT | WT | WT | Mut | Mut | Mut |
| **OXCO2** | CCI | IMDM | X | WT | WT | WT | WT | WT | X | X |
| **SNUC1** | ATCC | RPMI 1640 | WT | WT | WT | WT | WT | WT | WT | Mut |
| **SW48** | ICR | DMEM | Mut | WT | WT | WT | WT | WT | Mut | WT |

Key: Minimum Essential Media (MEME), Dulbecco's Modified Eagle Medium (DMEM), Iscove's Modified Dulbecco's Medium (IMDM), RPMI 1640 with Insulin 10 µg/ml, Hydrocortisone 50 µM, 1-thioglycerol 1.25g/ml (RPMI +-supp), Dulbecco's Modified Eagle Medium: Nutrient Mixture F-12 (DMEM/F12),American Tissue Culture Collection (ATCC), Public Health England (PHE), Japan Cell bank (JCB), Candiolo Cancer Institute Turin (CCI), ICR (institute of Cancer Research, Wild-type (WT), Mutant (Mut) data not available (X)

**Supplementary Table 1: List of *KRAS/NRAS/BRAF* WT CRC cell lines used:** RPMI 1640 was purchased from Gibco, UK. All other media were purchased from Sigma Aldrich, UK. Media were supplemented with 10% Fetal Bovine Serum (Sigma Aldrich, UK), unless otherwise specified, 1 mM L-Glutamine (25030-024, Gibco) and 1x minimum essential medium (MEM) non-essential amino acid solution (Sigma-Aldrich, M7145). All cell lines were cultured at 37 °C, 5% CO2. Data relating to each cell line’s mutation status (KRAS, NRAS, HRAS, BRAF, EGFR, PIK3CA, TP53, APC) were extracted from the Catalogue of Somatic Mutations in Cancer (COSMIC) and Cancer Cell Line Encyclopedia (CCLE) databases.
