# Supplementary material for: A phospho-proteomic study of cetuximab resistance in KRAS/NRAS/BRAFV600 wild-type colorectal cancer
Source: Cell Oncol (Dordr). 2021 Aug 30;44(5):1197–206. doi: 10.1007/s13402-021-00628-7 (PMC8516765; doi:10.1007/s13402-021-00628-7)
Supplement: Supplementary file 4 — Supplementary file4 (DOCX 13 kb) [file 13402_2021_628_MOESM4_ESM.docx]

| **Total Antibodies** | **Dilution** | **Product code** | **Source** |
| --- | --- | --- | --- |
| EGFR | 1:1000 | 4267 | Cell Signalling |
| GAPDH | 1:5000 | MAB374 | Merck Millipore |
| **Phospho-Antibodies** | **Dilution** |  |  |
| pEGFR (Tyr1068 | 1:500 | 3777 | Cell Signalling |
| pPRAS40(Thr 246) | 1:1000 | 13175 | Cell Signalling |
| pS6 (Ser 240/244) | 1:1500 | 5364S | Cell Signalling |
| Cleaved PARP (Asp214) | 1:1000 | 9541 | Cell Signalling |

**Supplementary Table 2:** Primary antibodies used for Western blotting experiments
